# Validating national trends in breast reconstruction hospital price variation: A detailed analysis of a state market

**DOI:** 10.1016/j.jpra.2026.05.037

**Published:** 2026-05-28

**Authors:** David Fronk, Augustine Deering, Andrew Ni, Roberto Martinez

**Affiliations:** a University of Texas Health Science Center at San Antonio, Long School of Medicine, San Antonio, TX 78229, USA; b University of Texas Health Science Center at San Antonio, Department of Plastic Surgery, 7703 Floyd Curl Dr, San Antonio, TX 78229, United States of America

**Keywords:** Breast reconstruction, Pricing, Price transparency, Health economics, Billing, Health policy, Access to care

## Abstract

The Hospital Price Transparency Rule requires hospitals to report rates for a selection of procedures. While previous studies have evaluated commercial payer-specific rates nationally, we analyzed variation in breast reconstruction charges in Texas. Our goal was to determine whether national patterns in procedure rate variation persist in smaller markets. For 17 Current Procedural Terminology (CPT) codes, we calculated within-hospital price ratios (WHRs) by dividing the highest commercial rate by the lowest rate for a given code at each hospital and across-hospital ratios (AHRs) by dividing the 90th percentile by the 10th percentile within each code. Averaged across all codes, Texas WHRs were 2.4 times their national counterparts, and Texas AHRs were 0.42 times their national counterparts. Texas and national WHRs showed no correlation across procedure codes (Spearman r = 0.154, p = 0.555), while Texas and national AHRs showed statistically significant correlation (Spearman r = 0.847, p < 0.001). Our results demonstrate that only some aspects of rate variation are consistent with national patterns, supporting the need for state-level research and advocacy for breast reconstruction patients and insurers.

## Introduction

Recent changes in healthcare price transparency have brought new visibility to the high variance in costs for breast reconstruction surgery in the United States. To facilitate more competitive healthcare markets, the Centers for Medicare & Medicaid Services (CMS) requires that hospitals publish price information that helps individuals and corporations compare prices for a given service.^1^ “Payer-specific rates” do not capture physician or anesthesia fees or necessarily reflect patient out-of-pocket costs in all circumstances.^2^ However, they serve as a proxy for what insurers expect to pay hospitals for a given service.^1^

Previous research utilized public prices to show the high variability of reconstructive breast surgery costs across the United States, with the same procedure commanding markedly different charges within and across hospitals.^3^ We aim to determine whether national patterns of breast reconstruction rate variation, as published in Rochlin et al., persist in smaller markets or if price variance partly emerges from unmeasured geographic characteristics. Based on the lack of geographic pattern in pricing nationwide, we hypothesized that variance in breast reconstruction prices within Texas will closely mirror national levels.^3^

## Methods

Using the Turquoise Health database, we extracted commercial payer-specific rates for the same 17 breast reconstruction CPT codes examined by Rochlin et al., restricted to Texas in 2023.^3^ We considered only private insurers, excluding Medicare, Medicaid, and self-pay. To account for regional differences in operational costs, we adjusted the per CPT price data using the Physician Fee Schedule Geographic Practice Cost Index (GPCI) provided by CMS for each code to capture local variation in costs, using the same methodology as the comparison national rates.^3^^,^^4^

We created within-hospital ratios (WHRs) and across-hospital ratios (AHRs) for each code.^3^ The WHR was calculated by dividing the highest commercial price by the lowest commercial price for each CPT code at each facility:

$$WHR_{X\;at\;Y}=\frac{Max\;price\;for\;CPT\;X\;at\;Hospital\;Y}{Min\;price\;for\;CPT\;X\;at\;Hospital\;Y}$$

We calculated the AHR for each code by aggregating all commercial prices reported by hospitals across Texas and dividing the 90th percentile price by the 10th percentile price for each code:

$$AHR_{X}=\frac{90th\;Percentile\;GPCI\;Adjusted\;Price\;for\;CPT\;X\;in\;Texas}{10th\;Percentile\;GPCI\;Adjusted\;Price\;for\;CPT\;X\;in\;Texas}$$

For each code, we calculated the hospital-level median negotiated price and took the median across hospitals.

## Results

Our final data set consisted of 193 hospitals reporting 29,697 rates across 17 types of breast reconstruction procedures. All Texas WHRs exceeded, while all Texas AHRs fell below their national counterparts. Averaged across all codes, Texas WHRs were 2.4 times their national counterparts (mean Texas WHR 4.9 vs mean national WHR 2.1), and Texas AHRs were 0.42 times their national counterparts (mean Texas AHR 4.2 vs mean national AHR 10.0). Texas and national WHRs showed no correlation across procedure codes (Spearman r = 0.154, p = 0.555, n = 17), while Texas and national AHRs showed statistically significant correlation (Spearman r = 0.847, p < 0.001, n = 17).

## Discussion

The elevation of Texas rate variation within individual hospitals (WHR) across all codes is inconsistent with our hypothesis that Texas charge data would mirror national data. Furthermore, there is no correlation between which codes have higher WHRs at the state and national levels. At a given hospital in Texas, one insurer could face a negotiated rate nearly five times higher than another, raising concerns for patient fairness and cost-sharing. The fact that Texas WHRs exceeded national benchmarks with an idiosyncratic pattern underscores the importance of regional analysis in understanding hospital pricing behavior and addressing potential equity concerns Table 1.

**Table 1:** Texas price variance versus national price variance per Rochlin et al.3.

		Geographically Adjusted Prices (USD)					
CPT Code	No. of Hospitals	Median (IQR)	Mean (SD)	Texas WHR (IQR)	National WHR^3^	Texas AHR	National AHR^3^
11,970	137	6097 (3677 - 7886)	6798 (4496)	4.59 (2.7 - 11.1)	*2.50*	4.7	*11.54*
19,316	135	5581 (3502 - 6521)	6594 (6377)	4.54 (2.3 - 9.2)	*1.85*	5.1	*10.58*
19,318	151	5842(3887 - 7513)	6523 (4312)	4.03 (2.1 - 9.7)	*2.37*	4.0	*8.95*
19,325	133	6921 (4551 - 9728)	7795 (5368)	5.89 (2.3 - 12.9)	*1.87*	5.0	*18.31*
19,328	141	3875 (2809 - 5206)	4419 (2635)	4.59 (3.0 - 13.6)	*2.80*	4.7	*8.29*
19,330	129	4020 (3014 - 5574)	4622 (3146)	4.59 (3.0 - 11.5)	*2.22*	4.7	*9.35*
19,340	135	6102 (3555 - 7824)	6946 (6111)	5.89 (2.6 - 13.0)	*2.38*	5.3	*11.87*
19,342	140	6874 (3967 - 9370)	8546 (7177)	6.91 (2.6 - 13.7)	*2.02*	5.6	*14.99*
19,350	130	4188 (3307 - 5698)	4654 (2650)	4.49 (2.3 - 6.7)	*2.09*	3.3	*7.16*
19,357	145	7717 (5002 - 15,021)	11,494 (12,823)	8.25 (3.0 - 16.3)	*2.11*	6.9	*14.79*
19,361	105	3563 (2481 - 6315)	5644 (5692)	5.89 (2.1 - 12.9)	*1.71*	4.8	*11.15*
19,364	95	4360 (3548 - 5652)	4733 (1871)	3.72 (2.5 - 6.5)	*1.88*	2.3	*4.45*
19,367	94	3564 (2746 - 5498)	4263 (2099)	4.36 (2.5 - 6.5)	*1.61*	3.1	*9.52*
19,368	92	3942 (3233 - 5622)	4511 (2004)	3.72 (2.2 - 6.5)	*1.92*	2.7	*4.78*
19,369	92	3837 (3028 - 5622)	4417 (2043)	3.82 (2.4 - 6.5)	*1.95*	2.8	*4.93*
19,370	130	4261 (3308 - 6011)	4981 (3321)	4.54 (2.4 - 6.8)	*2.18*	3.7	*8.65*
19,380	139	5753 (3895 - 7402)	6080 (3451)	4.05 (2.4 - 8.7)	*2.21*	3.1	*10.03*
*Note*: Code definitions can be found in Appendix A							

No. of Hospitals: Number of hospitals reporting data for the specific CPT/S code.

Median (IQR): Median price with Interquartile Range.

Mean (SD): Mean price with Standard Deviation.

Texas WHR (IQR): Within-Hospital Ratio with Interquartile Range calculated using Texas data.

National WHR: Within-Hospital Ratio calculated using national data.

Texas AHR: Across-Hospital Ratio calculated using Texas data.

National AHR: Across-Hospital Ratio calculated using national data.

In contrast, our hypothesis is more strongly supported in terms of price variation between hospitals within Texas (AHR). Texas demonstrates lower than the national between-hospital variation for each code. This is unsurprising, since hospitals in the same state likely have common regulations, insurance companies, and input costs. Nevertheless, the statistically significant correlation between codes indicates that the variance between hospitals follows similar patterns in Texas and nationally. This suggests certain codes are inherently more prone to variation between hospitals, regardless of the geographic markets.

## Conclusions

Our results partially support the hypothesis, with Texas within-hospital charge ratios for breast reconstruction codes averaging 2.4 times and Texas across-hospital charge ratios averaging 0.42 times their national counterparts. The correlation between Texas and national AHRs suggests that between-hospital charge patterns demonstrated by Rochlin et al. persist at the state level, while the absence of correlation in WHRs indicates that within-hospital charge dynamics differ from national trends. While national policy remains important given the federal government’s influence through CMS, our findings emphasize that state-level advocacy, research, and regulation are consequential in shaping hospital procedure markets and ultimately their fairness to patients.

## Disclosure

During the preparation of this work the authors used Claude Sonnet 4.6 for grammar checking and manuscript editing. After using this tool/service, the authors reviewed and edited the content as needed and take full responsibility for the content of the published article.

## Funding

None.

## Ethical approval

Not required.

## Declaration of competing interest

None declared.
